# Sequential electrodeposition of Cu–Pt bimetallic nanocatalysts on boron-doped diamond electrodes for the simple and rapid detection of methanol

**DOI:** 10.1038/s41598-021-92769-w

**Published:** 2021-07-13

**Authors:** Surinya Traipop, Abdulhadee Yakoh, Sakda Jampasa, Sudkate Chaiyo, Yuttanant Boonyongmaneerat, Joongjai Panpranot, Piyasan Praserthdam, Orawon Chailapakul

**Affiliations:** 1 Electrochemistry and Optical Spectroscopy Center of Excellence (EOSCE), Department of Chemistry, Faculty of Science, Chulalongkorn University, Bangkok, 10330 Thailand; 2 The Institute of Biotechnology and Genetic Engineering, Chulalongkorn University, Bangkok, 10330 Thailand; 3 Metallurgy and Materials Science Research Institute, Chulalongkorn University, Bangkok, 10330 Thailand; 4 Center of Excellence on Catalysis and Catalytic Reaction Engineering, Department of Chemical Engineering, Faculty of Engineering, Chulalongkorn University, Bangkok, 10330 Thailand

**Keywords:** Natural hazards, Health care, Chemistry, Materials science, Nanoscience and technology

## Abstract

In this work, a novel electrochemical sensor for methanol determination was established by developing a bimetallic catalyst with superiority to a monometallic catalyst. A Cu–Pt nanocatalyst was proposed and easily synthesized by sequential electrodeposition onto a boron-doped diamond (BDD) electrode. The successful deposition of this nanocatalyst was then verified by scanning electron microscopy and energy dispersive spectroscopy. The electrodeposition technique and sequence of metal deposition significantly affected the surface morphology and electrocatalytic properties of the Cu–Pt nanocatalyst. The presence of Cu atoms reduced the adsorption of other species on the Pt surface, consequently enhancing the long-term stability and poisoning tolerance of Pt nanocatalysts during the methanol oxidation process. This advanced sensor was also integrated with sequential injection analysis to achieve automated and high-throughput analysis. This combination can significantly improve the detection limit of the developed sensor by approximately 100 times compared with that of the cyclic voltammetric technique. The limit of detection of this sensor was 83 µM (S/N = 3), and wide linearity of the standard curve for methanol concentrations ranging from 0.1 to 1000 mM was achieved. Finally, this proposed sensor was successfully applied to detect methanol in fruit and vegetable beverage samples.

## Introduction

Recently, significant attention has been rightfully placed on the production of alternative nanomaterials, especially from an industrial perspective. The development of bimetallic nanomaterials is presumed to be an effective way to surpass the usual properties of traditional monometallic substances. Bimetallic materials are composed of two different metals that enhance their individual properties and exhibit several new characteristics from the collaborating metals. These synergistic properties may include the promotion of low-temperature oxidation, enhancement of the magnetic and optical properties, improvement of the oxidation resistance, and increased catalytic efficiency^1–3^. More importantly, bimetallic catalysts have been extensively employed in several fields. The mutual influence of different neighboring atoms can lead to catalytic behavior. Moreover, the addition of a secondary metal offers the potential to increase the activity, selectivity, and stability of catalysts for a particular reaction. Several bimetallic catalysts have been utilized for numerous purposes, such as environmental treatments, chemical synthesis, and petroleum refining processes^4–6^. Among various applications, we are particularly interested in exploiting bimetallic catalysts for the sluggish oxidative kinetic reaction of the direct electrochemical detection of methanol (MetOH).

Typically, MetOH itself is not toxic to animal cells. However, it is considered to be toxic to humans when ingested because it results in the accumulation of highly toxic metabolites. Alcohol dehydrogenase converts MetOH into formaldehyde, which is rapidly processed into formic acid. Acute intoxication of MetOH can cause central nervous system depression, vertigo, fatigue, blurred vision, blindness, and death^7,8^. In the electrochemical view, MetOH sensors can be categorized into two groups: enzymatic assays and nonenzymatic assays. For the enzymatic assay, a few electrochemical sensors have been reported that use alcohol dehydrogenase enzyme-modified electrodes^9,10^. Although the enzyme-based electrode provides a selective and sensitive response, the thermal and chemical instability of the enzyme usually hinders the functionality of this modified electrode. To avoid these issues, the use of the nonenzymatic electrochemical method based on the direct analysis of MetOH has become more appealing. For nonenzymatic assays, the electrochemical behavior of MetOH strongly relies on the catalytic activity of the catalyst. From this perspective, numerous catalytic metals (e.g., Pt, Ag, Au, and Cu) have been studied to improve the electrochemical oxidation of MetOH^11–13^. Among them, Pt has been recognized as the most powerful catalyst for the electrocatalytic oxidation of MetOH due to its ability to break the C–H bond^14^. However, various partial oxidation intermediates [carbon monoxide (CO)-containing species] can be formed during the MetOH oxidation process and strongly adsorb on Pt active sites. The loss of catalytic activity due to Pt catalyst poisoning is a major limitation for direct determination. To improve CO tolerance, Pt-based bimetallic catalysts have been widely studied^15–17^. By doping other atoms into Pt, several effects, such as lattice strain, surface ligands, geometric, and synergetic effects, promote their performance to complete MetOH oxidation. Interestingly, the modeling predictions have suggested Cu as a promising catalyst for MetOH oxidation^18,19^. The overpotential for Cu–Pt was significantly lowered compared to that of the other elemental Pt-based catalysts. Furthermore, Cu has attracted considerable attention not only due to its low cost but also because Cu itself can induce a lattice strain effect, thus overcoming the CO adsorption issue. Although bimetallic Cu–Pt for MetOH oxidation has been reported recently^20,21^, the bimetallic formation processes are still laborious and time-consuming, using several hazardous reagents.

Herein, a bimetallic Cu–Pt nanocatalyst-modified boron-doped diamond (Cu–Pt/BDD) electrode was proposed for MetOH detection. BDD was used as the electrode substrate in this work due to its excellent resistance to corrosion and good stability over time in the highly corrosive environment of MetOH oxidation^22^. Rapid and straightforward preparation of the bimetallic Cu–Pt catalyst through sequential electrodeposition was reported in this work. The order of metal deposition and the electrodeposition technique were found to be the key factors affecting the MetOH oxidation performance. Moreover, the automatic online system of sequential injection analysis (SIA) has been coupled with the electrochemical system for more precise control, thus enabling efficient analysis. To demonstrate the functionality of the proposed system, we performed a series of assays in real samples (fruit and vegetable beverages). The analysis results clearly justify the potential applicability of the sensor for routine testing in an accessible manner.

## Results and discussion

### Electrocatalytic activity toward MetOH oxidation in an alkaline medium

MetOH oxidation was studied using CV in a 1 M MetOH solution. Initially, the effect of the electrolyte (NaOH, KHCO_3_, PBS, K_2_SO_4_, and H_2_SO_4_) was investigated using a Pt-modified BDD (Pt/BDD) electrode. It has been reported in the literature that alkaline media can improve MetOH oxidation^14^. As presented in Supplementary Fig. S1, it can be clearly observed that the use of NaOH can substantially increase the electrocatalytic activity for MetOH oxidation. This enhanced sensitivity can be explained by the oxygenated NaOH, which promotes the oxidation of carbonaceous intermediates (such as carbon monoxide, CO) adsorbed on the Pt surface, thereby enhancing the sensitivity toward MetOH oxidation. Normally, the electrochemical oxidation of MetOH is driven by a six-electron transfer as Eq. (1). This reaction is sluggish without catalysts.

1 $${\text{CH}}_{{\text{3}}} {\text{OH}}~ + ~{\text{H}}_{{\text{2}}} {\text{O}}~ \to ~{\text{CO}}_{{\text{2}}} ~ + ~{\text{6H}}^{ + } ~ + ~{\text{6e}}^{ - } .$$

The electro-oxidation of MetOH on Pt catalyst can be clarified by Eqs. (2) and (3)^23^.

2 $${\text{CH}}_{{\text{3}}} {\text{OH}}~ \to ~\left( {{\text{INTMD}}} \right)~ \to ~{\text{CO}}_{{\text{2}}} ,$$

3 $${\text{Pt}} - \left( {{\text{CO}}} \right)_{{{\text{ad}}}} ~ + ~{\text{Pt}} - \left( {{\text{OH}}} \right)_{{{\text{ad}}}} ~ + ~{\text{2Pt}}~ \to ~{\text{CO}}_{{\text{2}}} ~ + ~{\text{H}}^{ + } ~ + ~{\text{e}}^{ - } .$$

MetOH is initially adsorbed on the surface of Pt catalyst to form the adsorbed intermediates and further oxidized to CO_2_. The strongly adsorbed species, Pt–(CO)_ad,_ can be formed during the oxidation process. When Pt–(CO)_ad_ accumulates on the Pt active sites, the kinetic rate of MetOH oxidation is decelerated and Pt catalyst becomes poisoned. Pt–(OH)_ad_ which easily formed in alkaline solution activates oxidation of Pt–(CO)_ad_ to CO_2_ and releases Pt active sites again as seen in Eq. (2). Although Pt–(OH)_ad_ also forms in acidic media, the current of MetOH oxidation does not improve because Pt–(OH)_ad_ can be formed at higher potential as seen in the 2nd forward oxidation at 0.8 V in Supplementary Fig. S1e. Therefore, NaOH will be further used throughout the experiment.

**Figure 1:**
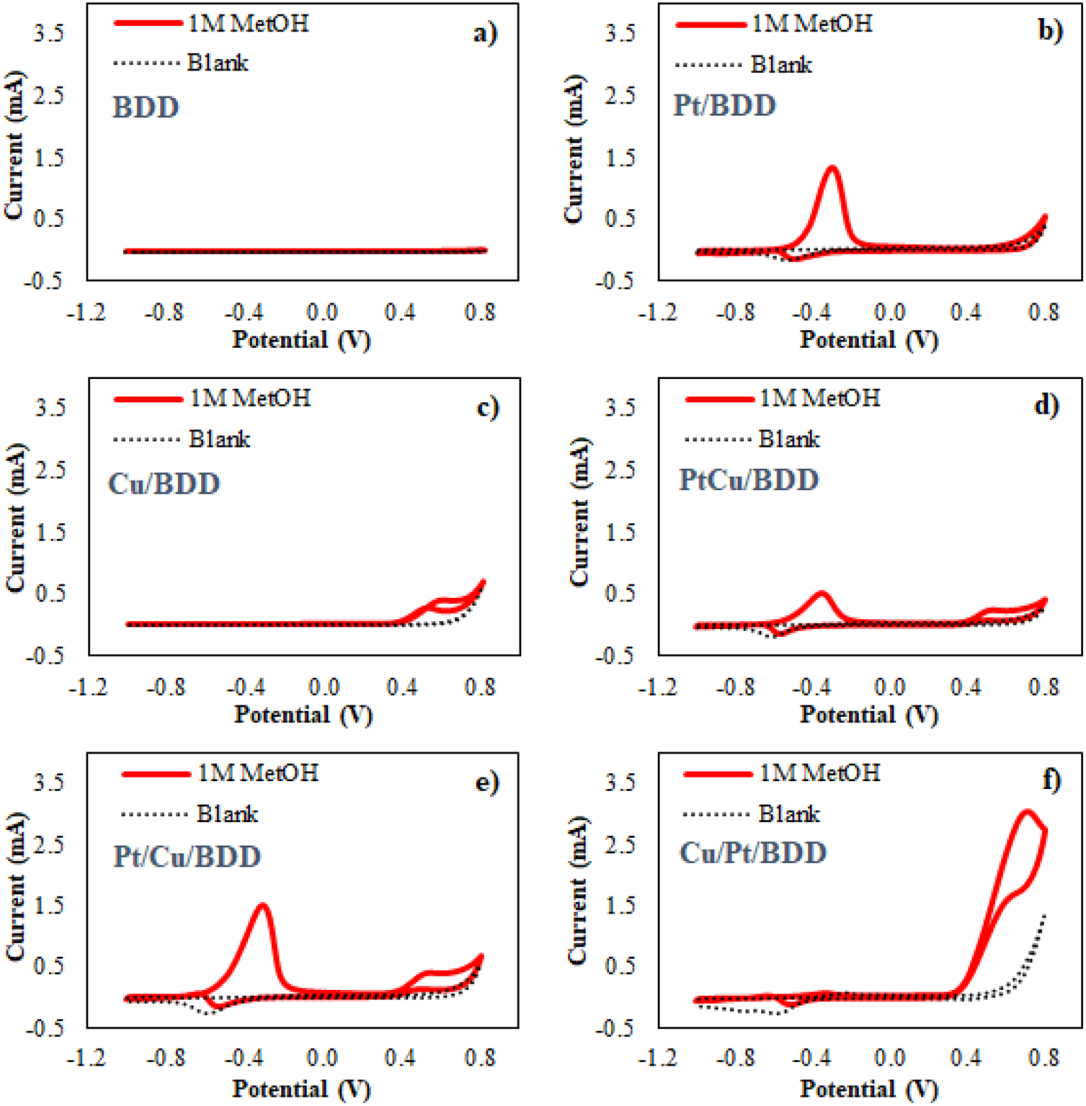
Cyclic voltammogram of 1 M MetOH in 0.1 M NaOH on the (**a**) bare BDD electrode compared with the modified electrodes: (**b**) Pt/BDD, (**c**) Cu/BDD, (**d**) PtCu/BDD, (**e**) Pt/Cu/BDD, and (**f**) Cu/Pt/BDD electrodes. Scan rate: 100 mV/s. All electrodes were prepared via multiple-step deposition technique.

Next, the effect of the Pt and Cu nanocatalysts was studied by scanning from − 1.0 to 0.8 V vs Ag/AgCl at a rate of 100 mV/s. The results showed that the oxidation of MetOH was negligible on the bare BDD electrode within the studied region (Fig. 1a), whereas the well-defined peak of MetOH oxidation was clearly observed at a relatively low potential (− 0.32 V vs Ag/AgCl) on the Pt/BDD electrode, as illustrated in Fig. 1b. Note that the reduction peak in the blank solution could be ascribed to the reduction of platinum oxides (Pt–O_x_)^24^ after depositing 1 mM K_2_[PtCl_4_] on the BDD surface via a multiple-step deposition technique (see the potential waveform in Supplementary Fig. S2). For the Cu-modified BDD (Cu/BDD) electrode, the small oxidation peak of MetOH appeared at a more positive potential of 0.60 V vs Ag/AgCl, as shown in Fig. 1c. These observations demonstrated that both modified electrodes exhibited electrocatalytic properties toward MetOH oxidation. Moreover, the higher current response on the Pt/BDD electrode indicated that the electrocatalytic activity of Pt toward MetOH oxidation was superior to that of Cu. However, the strong adsorption of oxygenated species on the Pt surface was observed, resulting in an inactive surface after forward oxidation. This can be monitored by the disappearance of the backward oxidation on the Pt/BDD electrode, which is related to the freshly chemisorbed MetOH on the free metal surface^25^. Even though the adsorption of oxygenated species can improve the CO tolerance level, the strong adsorption inhibited the renewability of the electrode surface. To prevent Pt catalyst poisoning from the adsorbed species, doping Pt with other atoms is one of the solutions.

Consequently, a bimetallic Cu–Pt-modified electrode was investigated under the same conditions as the individual deposition. We also evaluated the influence of the different electrodeposition procedures (simultaneous and sequential electrodeposition) for bimetallic nanocatalyst formation on MetOH oxidation. For the simultaneous electrodeposition of Cu–Pt (PtCu/BDD), two oxidation peaks were observed at identical positions, in which MetOH was oxidized on the individual deposited electrodes, as shown in Fig. 1d. We then investigated the modified electrode prepared via a sequential electrodeposition procedure. Figure 1e,f show the CV curves of MetOH oxidation recorded using Pt/Cu/BDD (an electrodeposition of Cu on the BDD electrode followed by Pt electrodeposition) and Cu/Pt/BDD (an electrodeposition of Pt on the BDD electrode followed by Cu electrodeposition) electrodes, respectively. Figure 1e shows that, for the Pt/Cu/BDD, the comparable sensitivity compared to that using individual Pt or Cu modified electrodes was obtained. Two clear peaks corresponding to their catalytic metals were observed. Additionally, further Pt deposition on top of Cu/BDD surface (by increasing the Pt concentration and deposition time) is still unable to enhance the sensitivity and combine these two separated peaks. However, a dramatically enhanced signal (with backward oxidation) was clearly observed at 0.71 V vs Ag/AgCl when Cu/Pt/BDD electrode was utilized (Fig. 1f). This phenomenon could arise from the nature of Pt atoms, which normally form clusters by electrodeposition^26,27^. This is probably a factor that makes it difficult to achieve full coverage the Pt atoms over Cu atoms. Full coverage of the second atoms provided more active sites, which MetOH can be oxidized at the same region, leading to higher sensitivity in the case of Cu/Pt/BDD electrode. In addition, the presence of Cu atom has been reported to dramatically decrease the d-band center energy, ε_d_, of Pt compared with other metal atoms^28^. The lower energy decreases the adsorption tendency of Pt; therefore, the adsorption can be suppressed on bimetallic Cu/Pt/BDD electrode, leading to more stability than Pt/BDD electrode as seen in Supplementary Fig. S3. Hence, the sequential electrodeposition of Cu on top of the Pt layer (Cu/Pt/BDD) exhibits a synergistic effect toward MetOH oxidation with regard to electrode poisoning prevention.

### Surface morphologies of the modified electrodes

**Figure 2:**
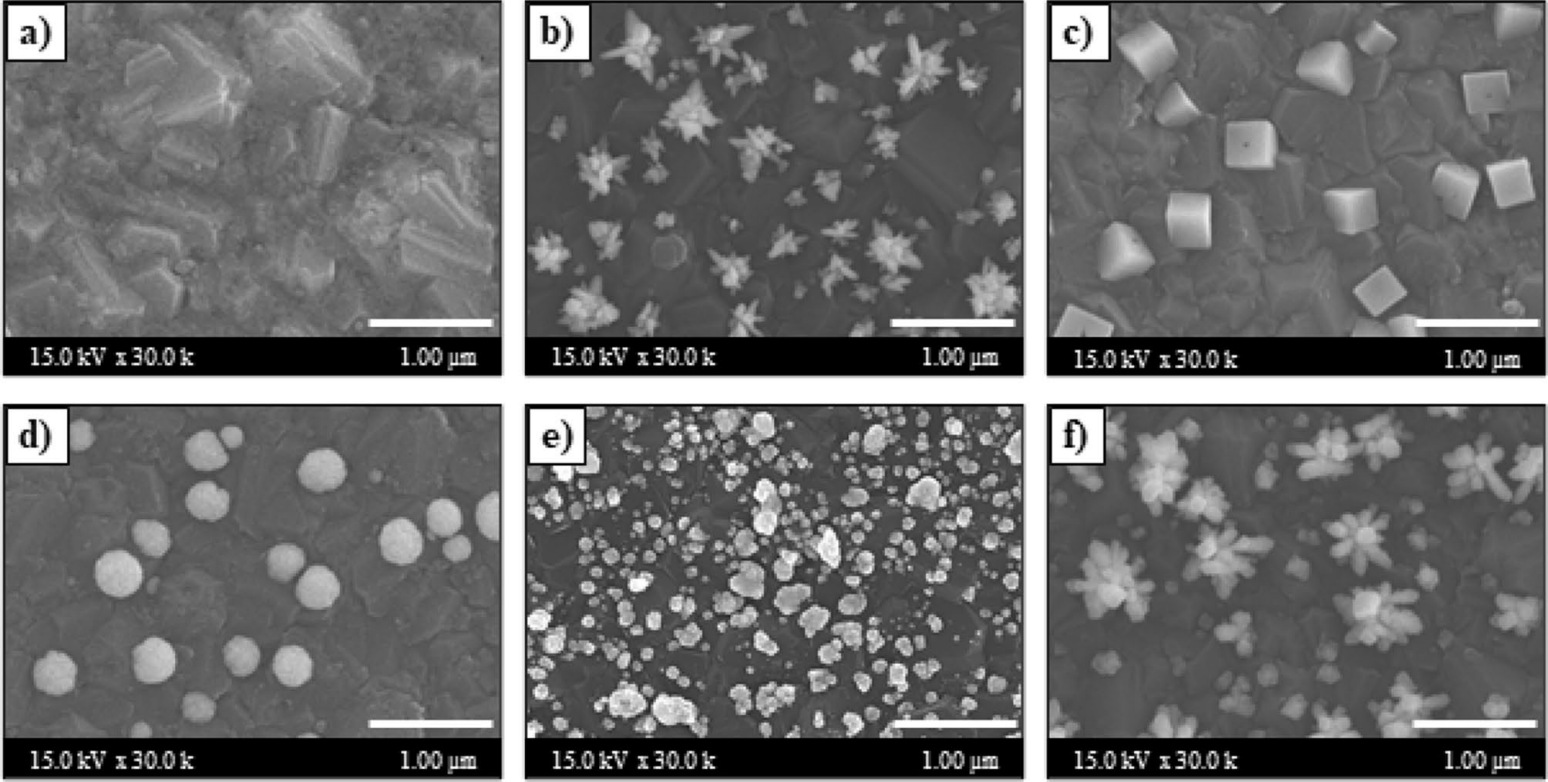
SEM images with ×30,000 magnification of (**a**) BDD, (**b**) Pt/BDD, (**c**) Cu/BDD, (**d**) PtCu/BDD, (**e**) Pt/Cu/BDD, and (**f**) Cu/Pt/BDD electrodes prepared via multiple-step deposition technique.

The morphologies of the bare BDD, Pt/BDD, Cu/BDD, PtCu/BDD, Pt/Cu/BDD, and Cu/Pt/BDD electrodes prepared via multiple-step deposition were characterized using SEM (Fig. 2a–f). The morphology of the bare BDD electrode shown in Fig. 2a revealed the roughness of the BDD electrode surface owing to boron-doped diamond crystal growth on the silicon support. When Pt was electrodeposited on the BDD electrode, the flower-like structure of Pt particles was observed on the BDD electrode (Pt/BDD), as shown in Fig. 2b. The Cu deposition formed a cubic structure on the BDD electrode (Cu/BDD) (Fig. 2c). In cases of bimetallic catalysts, the effect of electrode preparation procedures (simultaneous and sequential deposition) on the nanocatalyst morphologies was studied. For the simultaneous deposition of Pt and Cu, the mixture of Pt and Cu can produce spherical particles on the BDD electrode (PtCu/BDD), as illustrated in Fig. 2d. Figure 2e,f show the images obtained from sequential electrodeposition. The morphology of the Pt/Cu/BDD electrode was irregular particles (Fig. 2e). On the other hand, the morphology of the Cu/Pt/BDD electrode showed the growth of Cu particles on the flower-like Pt base (Fig. 2f). The flower-like structures were still observed with larger spines of the flower-like morphology. This three-dimensional structure provides a favorable surface area and active center for electrocatalysis due to their abundant atomic steps, edges, and corner atoms in their 3D structure, resulting in promoted MetOH oxidation^20^.

**Figure 3:**
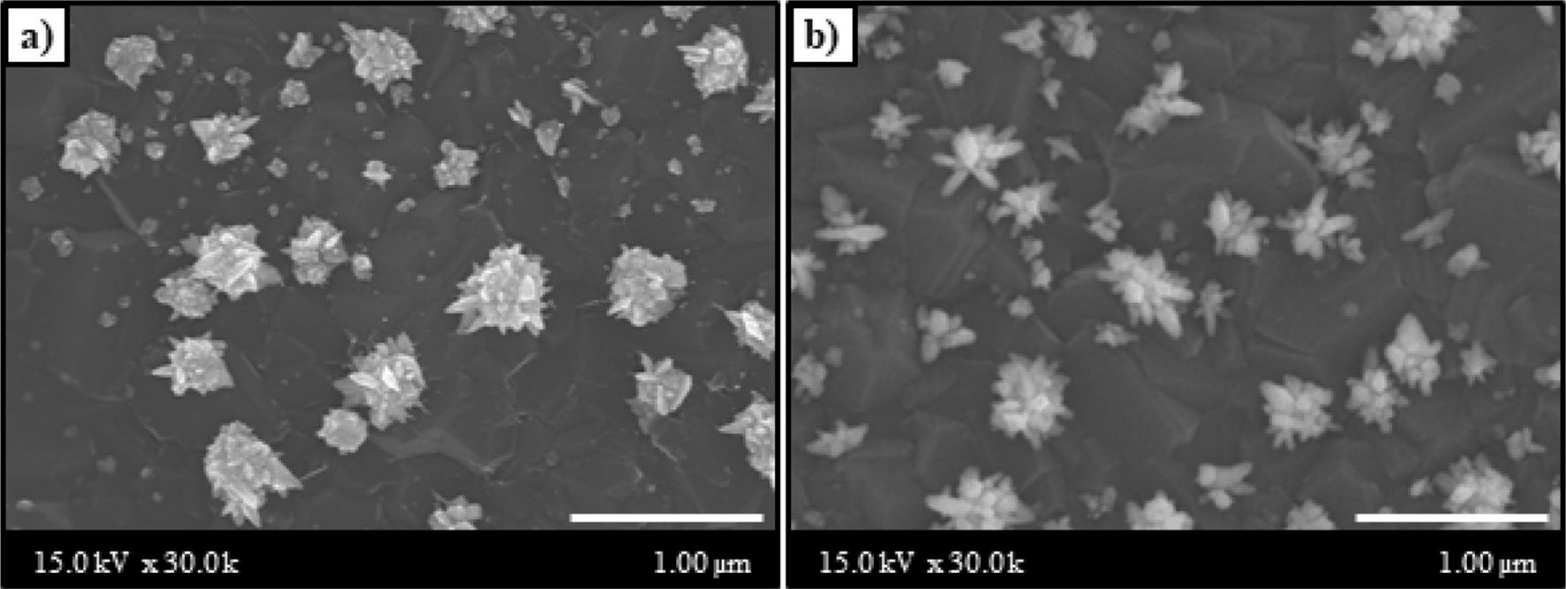
SEM images of Pt/BDD electrode fabricated by (**a**) one-step and (**b**) multiple-step electrodepositions.

Moreover, as Pt electrodeposition plays an important role in MetOH oxidation, we studied the influence of the deposition techniques (one-step deposition and multiple-step deposition) on the morphologies and current responses of the modified electrode. The one-step deposition was performed by applying a static reduction potential during the deposition step, while the multiple-step deposition applied a reduction potential for a fixed period, followed by a relaxation potential in repeated cycles (Supplementary Fig. S2a,b). The SEM images of the Pt/BDD surface prepared with multiple-step and one-step electrodeposition are shown in Fig. 3. Both Pt/BDD electrodes (prepared with different techniques) revealed the presence of Pt microclusters arising from the agglomeration of several Pt particles to form a flower-like morphology. However, multiple-step deposition caused a better Pt dispersion on the BDD surface. Moreover, many spines of flower-like morphology were clearly observed on the multiple-step Pt/BDD electrode, leading to more active surface area to react with MetOH. This result can be explained by the increase in the number of new nuclei for Pt deposition on the BDD support and particle growth on the Pt nucleus due to the pulse of the potential applied in the multiple-step deposition. Hence, the multiple-step deposition was suitable for the electrodeposition of Pt on the BDD electrode; the result was in agreement with previous studies^22^. Additionally, the voltametric CV responses (Supplementary Fig. S2c) indicate that the multiple-step deposition provided an anodic current response that was higher than that of the one-step deposition. In contrast, one-step or multiple-step deposition of the Cu/BDD electrode showed an identical current response (Supplementary Fig. S2d). Consequently, multiple-step electrodeposition was performed for Pt deposition on the BDD electrode, whereas one-step electrodeposition was used for Cu deposition on the Pt/BDD electrode in further studies.

**Figure 4:**
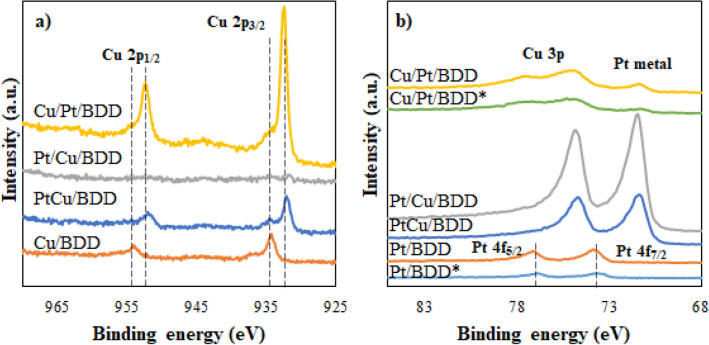
XPS spectra for (**a**) Cu 2p and (**b**) Pt 4f of the modified electrodes [Pt/BDD, Cu/BDD, PtCu/BDD, Pt/Cu/BDD, and Cu/Pt/BDD fabricated by multiple-step deposition; Pt/BDD* and Cu/Pt/BDD* fabricated by one-step deposition of Pt and Cu, respectively].

To verify the chemical state and electronic structure of each element, XPS analysis was performed. The XPS spectra of Cu 2p was illustrated in Fig. 4a. For Cu/BDD electrode, two peaks of Cu 2p_1/2_ and 2p_3/2_ positioned at 954.1 and 934.4 eV were observed, corresponding to Cu^0^/Cu^+^^29^. A negative shift of ~ 1.8 eV was observed for Pt-contained electrodes, indicating an alloy formation between Cu and Pt. Nonetheless, Cu 2p disappeared in the XPS spectrum of the Pt/Cu/BDD electrode. This effect could be described by the extensive coverage of the endmost deposition of Pt atoms on the electrode surface (corresponding with SEM image in Fig. 2), thereby hindering the X-ray penetration of the initial deposition of Cu atoms. For the Pt 4f spectra (Fig. 4b), the corresponding Pt peaks of Pt/BDD electrode were observed at 77.2 and 73.8 eV for Pt 4f_5/2_ and Pt 4f_7/2_, respectively, which suggest the presence of PtO_2_/PtO species on the surface. In the presence of Cu atom, the shift of binding energies of the Pt 4f_5/2_ and Pt 4f_7/2_ to 74.7 and 71.5 eV indicated that most of the Pt species are presented as metallic Pt^0^ and/or Pt–Cu alloy. Moreover, the electrodeposition technique (one- or multiple-step) does not affect the chemical state and electronic properties of Pt and Cu (such as Cu/Pt/BDD and Cu/Pt/BDD*). Anyhow, comparing the bimetallic electrodes between the PtCu/BDD (simultaneous deposition) and the Cu/Pt/BDD* (sequential deposition), it was observed that the Cu/Pt/BDD* exhibited much higher intensities of the peaks attributing to Cu 2p and an overlapping peak of Cu 3p at 74.7 and 77.3 eV with Pt 4f_5/2_. It has been suggested the distribution and electronic effect between Pt and Cu atoms were enhanced with increasing Cu content^30^. The amount and ratio of Cu and Pt were also presentenced in Supplementary Table S2. The results clearly revealed that the appearance of Pt base improved the electrodeposition of Cu on the electrode surface. In addition, the presence of Pt and Cu on the Cu/Pt/BDD electrode fabricated under the optimized conditions was confirmed by energy dispersive spectroscopy (EDS). The EDS mapping of elements in Supplementary Fig. S4 revealed the presence of Pt and Cu atoms on the Cu/Pt/BDD electrode with an atomic ratio of 60:40% Pt:Cu atoms.

### Electrochemical characterization of BDD, Pt/BDD, Cu/BDD, and Cu/Pt/BDD electrodes

A ferro/ferricyanide ([Fe(CN)_6_]^3−/4−^) redox couple system was employed for the electrochemical characterization. For the electrical conductivity, the electron transfer property was investigated by means of electrochemical impedance spectroscopy (EIS). The measurements were performed on the bare BDD, Pt/BDD, Cu/BDD, and Cu/Pt/BDD electrodes in the presence of 1 mM [Fe(CN)_6_]^3−/4−^ in 0.05 M KCl solution. The Nyquist plots shown in Supplementary Fig. S5 were fitted with the equivalent circuit. The semicircle diameter represents the charge transfer resistance (R_ct_), and the linear part corresponds to diffusion. R_ct_ values were found to be 49.0, 19.2, 16.9, and 14.8 kΩ for the bare BDD, Pt/BDD, Cu/BDD, and Cu/Pt/BDD electrodes, respectively. The lower resistance indicates higher conductivity. As a result, the conductivity was the lowest on the bare BDD electrode but increased after modification with Pt and/or Cu. For individual depositions, the Cu/BDD electrode had better conductivity than the Pt/BDD electrode. This is related to the fact that the electrical conductivity of Cu metal is higher than that of Pt metal. Interestingly, Cu/Pt/BDD showed the highest conductivity, which could be the synergistic result of Cu and Pt. Moreover, the R_ct_ value is related to the heterogeneous electron transfer rate constant (K_et_)^31^. The calculated K_et_ values for the bare BDD, Pt/BDD, Cu/BDD, and Cu/Pt/BDD electrodes were 0.66 × 10^–5^, 1.69 × 10^–5^, 1.92 × 10^–5^, and 2.19 × 10^–5^ cm/s, respectively. These results demonstrated the easy and fast electron transfer process on the modified Cu/Pt/BDD electrode.

Furthermore, the electroactive surface area (A_eff_) was determined by CV at various scan rates (Supplementary Fig. S6). Pt- and/or Cu-modified BDD electrodes significantly improved the peak current and reduced the peak separation of [Fe(CN)_6_]^3−/4−^ compared with the bare BDD electrode. The peak current obtained from all studied electrodes was proportional to the scan rate. According to the Randles–Sevcik equation, A_eff_ can be calculated by the relation of the square root of the scan rate^32^. The A_eff_ values of the bare BDD, Pt/BDD, Cu/BDD, and Cu/Pt/BDD electrodes were calculated to be 0.095, 0.097, 0.205 and 0.303 cm^2^, respectively. The studied electrochemical characteristics are summarized in Supplementary Table S1. The results clearly revealed that both the electrical conductivity and effective surface area were improved after metal electrodeposition on the BDD electrode. Cu played a superior role in the electrochemical characteristics than Pt because of its higher conductivity. In addition, sequential deposition of Pt and Cu provided the best result, which was attributed to the large surface area, high conductivity, and synergistic effect between Cu and Pt on the BDD electrode.

### Electrodeposition optimization

**Figure 5:**
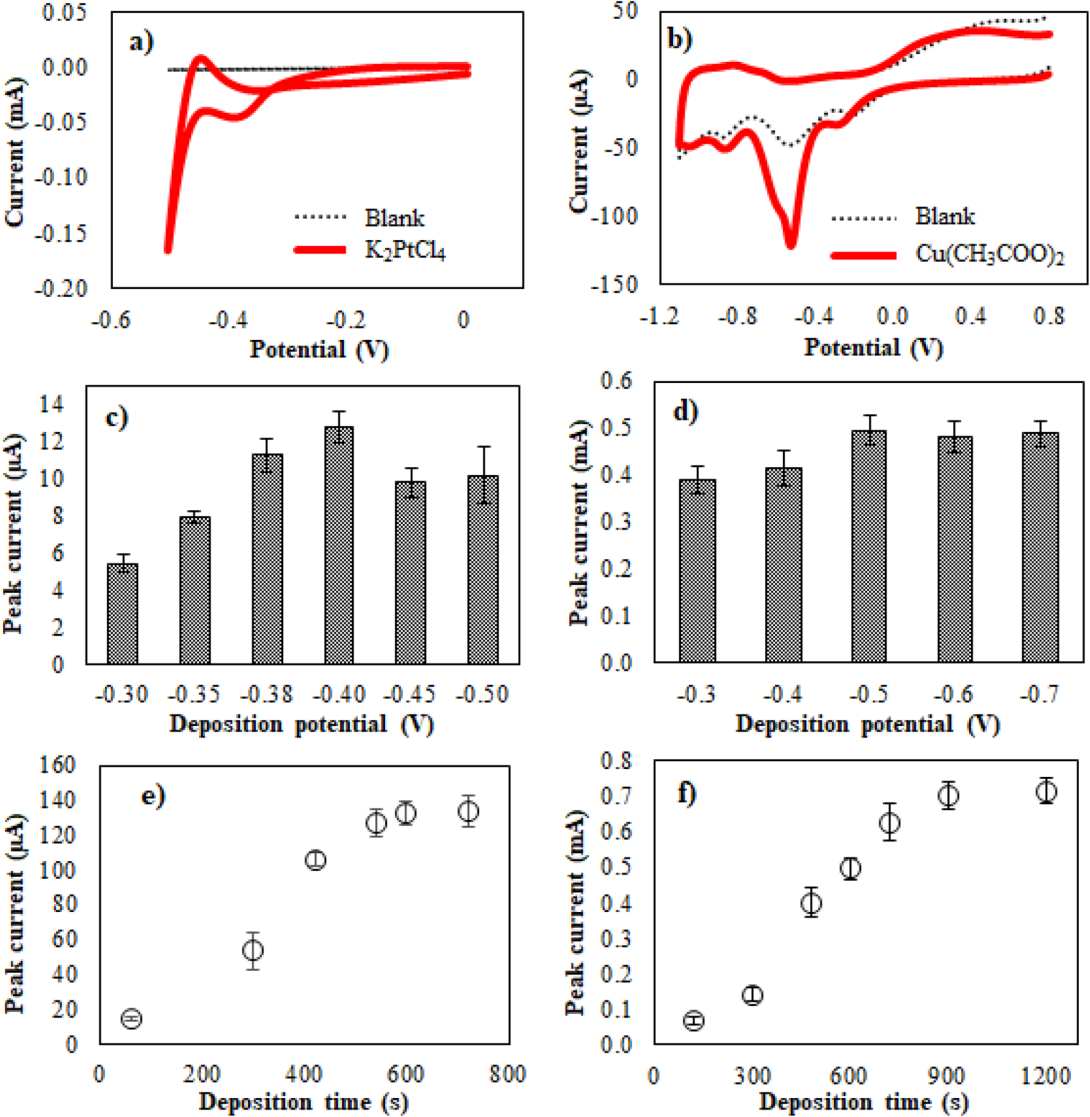
Cyclic voltammograms of (**a**) Pt reduction on BDD electrode, and (**b**) Cu reduction on Pt/BDD electrode; optimization of deposition potential of (**c**) Pt and (**d**) Cu; optimization of deposition time of (**e**) Pt and (**f**) Cu.

To fabricate the Cu/Pt/BDD electrode with the best electrocatalytic properties, the electrodeposition parameters were optimized. First, the reduction potentials of Pt and Cu were evaluated by CV, as shown in Fig. 5a,b, respectively. The cyclic voltammogram recorded for electrodeposition of Pt on the BDD electrode in a 0.1 M H_2_SO_4_ solution containing 1 mM K_2_[PtCl_4_] is shown in Fig. 5a. In this case, the potential scan started from 0.0 V to − 0.5 V vs Ag/AgCl to complete a cycle at a scan rate of 100 mV/s. The reduction potential of Pt on the BDD electrode was observed at − 0.38 V owing to the reduction of Pt(II) to Pt(0). In the case of Cu reduction on the Pt/BDD electrode, the potential was scanned from 0.8 V to − 1.0 V vs Ag/AgCl in a 0.1 M sodium acetate solution containing 1 mM Cu(CH_3_COO)_2_ with the same scan rate. The reduction peak of Cu on the Pt/BDD electrode appeared at − 0.5 V vs Ag/AgCl (Fig. 5b). Consequently, the deposition potentials in the ranges of − 0.3 to − 0.5 V vs Ag/AgCl and − 0.3 to − 0.7 V vs Ag/AgCl were investigated for Pt and Cu electrodeposition, respectively. Cyclic voltammograms revealed that the electrodeposition occurs with a slower rate at the initial onset of reduction peaks (− 0.3 V) than those of more negative potentials because of the higher kinetic driving force. Therefore, the deposition potential is further optimized.

The variations in deposition potential affecting the oxidation of 0.1 M MetOH are shown in Fig. 5c,d. MetOH oxidation on the Pt/BDD electrode (Fig. 5c) increased when the deposition potential was increased to a more negative value from − 0.3 to − 0.4 V vs Ag/AgCl and provided the maximum current at − 0.4 V vs Ag/AgCl, which can be attributed to the increase in the amount of Pt deposited on the electrode surface. The same result was also observed for Cu electrodeposition (Fig. 5d). The highest current was obtained at the peak potential (− 0.5 V vs Ag/AgCl) of Cu reduction on Pt/BDD electrode. According to Faraday's second law of electrolysis, the electrochemical equivalent of an element is directly proportional to its equivalent weight. One Faraday (96,500 C) liberates 1 g equivalent of the substance. Therefore, amounts of deposited metals can be calculated from their electrodeposition profiles and Faraday's second law application. The amounts of Pt atoms on BDD electrode and Cu atoms on Pt/BDD electrode at various deposition potentials in the optimization (Fig. 5c,d) were calculated and shown in Supplementary Table S3. The amount of Pt and Cu deposited on BDD or Pt/BDD electrode increased as applying more negative potential according to increasing kinetic driving force. Nonetheless, the estimated amount of Pt deposited at − 0.45 V, which reduction of H^+^ initially formed H_2_, might be inaccurate due to H_2_ interruption. This phenomenon was obviously observed at deposition potential of − 0.5 V which reduction current increase dramatically as seen in Fig. 5a. The deposition profile of − 0.5 V deposition was oscillated as H_2_ bubbles forming, leading to a decrease in the electrochemical equivalent and increase variable. Consequently, the MetOH oxidation current dramatically decreased at more negative potentials than − 0.4 V vs Ag/AgCl. This was a result of the bubbles interrupting Pt electrodeposition on the BDD electrode via hydrogen evolution as described before, which easily occurred at high negative potential or in strongly acidic media. Afterward, the deposition time was studied at the optimized deposition potential. The MetOH oxidation current increased and remained stable after the deposition time reached 600 s for Pt and 900 s for Cu, as shown in Fig. 5e,f, respectively. Therefore, Pt was electrodeposited on the BDD electrode at − 0.4 V vs Ag/AgCl for 600 s. Then, Cu was subsequently electrodeposited on the Pt/BDD electrode at − 0.5 V vs Ag/AgCl for 900 s.

### Analytical performance

**Figure 6:**
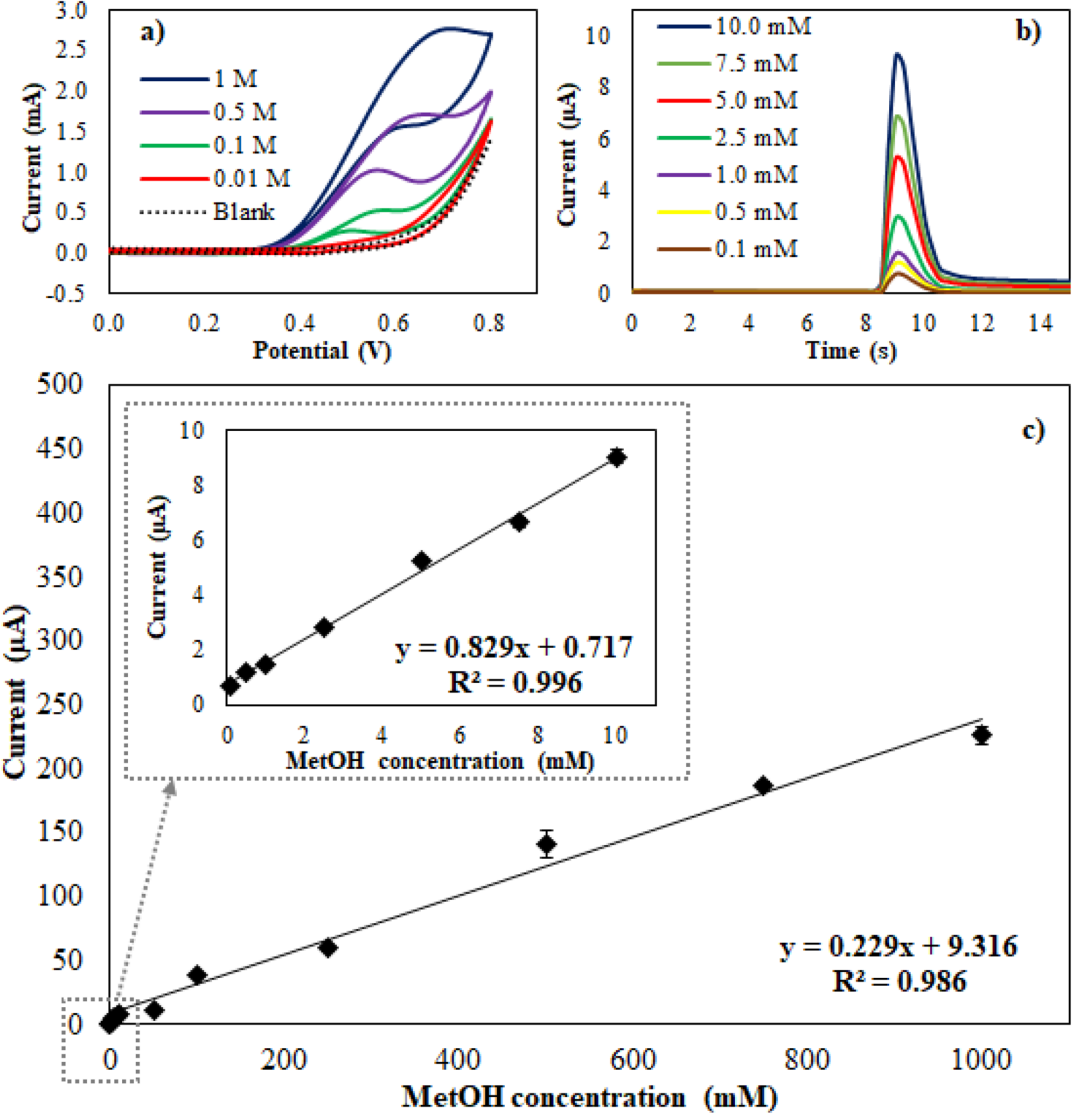
(**a**) Cyclic voltammograms of 0.1 M NaOH containing different concentrations of methanol varied form 0.01–1 M on Cu/Pt/BDD electrode. (**b**) Chronoamperograms of methanol detection in 0.1 M NaOH in the presence of methanol concentrations ranging from 0.1 to 10 mM using Cu/Pt/BDD electrode coupled with SIA system under the optimized conditions: 0.50 V initial potential for 15 s. (**c**) Calibration curve obtained from chronoamperometry for the methanol determination (0.1–1000 mM) using Cu/Pt/BDD electrode coupled with SIA system in an alkaline solution. Inset showed the calibration plot for 0.1–10 mM methanol.

The modified Cu/Pt/BDD electrode was applied to detect serial dilutions of MetOH. As shown in Fig. 6a, the oxidation peak was proportional to the MetOH amount; however, the current response of a low MetOH concentration (lower than 0.1 M) was rarely observed using CV. To achieve higher sensitivity, amperometry was then implemented in this work, as it provides much more current sensitivity than CV. Furthermore, to widen applications and to obtain more efficient analyses, the automated flow-based analytical system SIA was integrated with this detection system (Supplementary Fig. S7). Figure 6b shows the amperometric response of the Cu/Pt/BDD electrode coupled with the SIA technique for various MetOH concentrations (0.1–10 mM) under the optimized parameters. Even though the MetOH concentration was lower than 10 mM, a well-defined peak was observed, and the current response linearly increased with MetOH concentration. As shown in Fig. 6c, a break of the calibration curve was found at approximately 10 mM of the tested MetOH, implying that there were two linear ranges for MetOH determination explored in this work. The attained linearities were from 0.1 to 10 mM and from 10 to 1000 mM with detection sensitivities of 0.829 and 0.229 µA/mM, respectively. The correlation coefficients of the linear relations were 0.996 and 0.986 for the first and second regions of straight lines, respectively. The break in the calibration curve could be from the formation of a submonolayer and a monolayer of MetOH on the electrode surface at lower or higher concentrations, respectively^33^. The limit of detection (LOD) (S/N = 3) was calculated to be 83 µM for the low region of the calibration plot.

In addition, the performance of the fabricated Cu/Pt/BDD electrode coupled with the SIA system was compared with other works. As summarized in Table 1, the LOD obtained by the proposed method was comparable to that of the enzymatic system. A wide linear range was observed, which is suitable for various applications. The sensitivity at low MetOH concentrations was high enough to classify trace MetOH levels. The results indicated that the proposed method could be applied for MetOH detection with a wide dynamic range, high sensitivity, and low detection limit. Moreover, the use of enzymes, reducing agents, hazardous chemicals, and complex processes are dispensable in this work.

**Table 1:** Comparison of the analytical performance of other modified electrodes in the literature reports.

Electrode	LOD (µM)	Linear range (mM)	Sensitivity (µA/mM)
GA/G/AOx/PNR/AuNP/MWCNT/SPCE^10^	100.8	0.3359–1	0.509
PdNPs@SBA-15-Nafion/GCE^34^	12	0.02–1	90.5
Ferricyanide/rGO/Chitosan/Pt^35^	0.012	1–7	0.479
SE/Pt/GC^36^	100	0.25–10; 50–10,000	0.014; 0.004
Cu/Pt/BDDE (This work)	83	0.1–10; 10–1,000	0.829; 0.229

### Repeatability, reproducibility, stability, and interference study

The electrochemical response of 10 mM MetOH in 0.1 M NaOH was examined by five independently fabricated Cu/Pt/BDD electrodes to evaluate the reproducibility of the proposed method. It was found that the response had acceptable reproducibility with a relative standard deviation (RSD) of 3.2% (Supplementary Fig. S8). For repeatability, 10 successive measurements were performed by employing the same Cu/Pt/BDD electrode, and an extremely low %RSD was attained (~ 3.7%). This result indicated the satisfactory reusability of the developed MetOH sensor. The storage stability of Cu/Pt/BDD electrode was additionally investigated. The modified electrode was stored in a desiccator at room temperature, and the results revealed that after 2 weeks, the current responses for MetOH remained at 92% of the original signal. Thus, it can be summarized that the proposed sensor has good stability for up to 2 weeks. However, the peak current tended to decrease (lower than 90% compared with its initial signal) after a longer storage time was applied (data not shown). The decrease in the current response might be from the poisoning of the sensor surface caused by oxygen in the air, which consequently resulted in the inhibition of an active area on the electrode surface. Thus, to extend its shelf-life, the fabricated sensor should be kept in an oxygen-free environment or N_2_ atmospheric conditions. Based on the results described here, the repeatability, reproducibility, and stability of this sensor were acceptable for MetOH detection.

In addition, the selectivity of this method was evaluated by testing a solution of 1 mM MetOH in the presence of potentially interfering compounds, including sucrose, glucose, NaCl, formic acid, ascorbic acid, propanol, ethanol, and starch. The results demonstrated that the presence of interferences resulted in a bit changing in background electrolyte, leading to background current raised by sample injection. Therefore, the standard addition method was introduced to correct the background. By the correction, all studied interferents could be subtracted with no significant interference (signal change < 5%) in the determination of MetOH (Supplementary Fig. S9). For the detection at a low level of MetOH, the interference by other alcohols, which need more electron transfer, was not significantly impacted overall performance. Moreover, the tested interferents did not interfere with MetOH oxidation, although the spiked interferents were added 10 times higher than the previous report^35^. It can therefore be concluded that this proposed sensor has high selectivity and specificity for MetOH detection.

### Cu/Pt/BDD electrochemical sensor coupled with SIA for real sample analysis

The human diet is a source of MetOH, especially from plant products. In plants, macromolecules, including DNA and proteins, are demethylated into MetOH. Moreover, cell wall pectin is demethylesterified by pectin methylesterase (PME), which is the main source of MetOH in plants. Using aspartame as a synthetic nonnutritive sweetener causes an increase in MetOH levels because of methyl ester degradation^37^. Recently, drinking juices from fruit and vegetables has gained increasing attention for nutrition balance and health improvement. Low doses of MetOH may be ingested from fruit, vegetables, fermented beverages, and aspartame, an artificial sweetener, which breaks down to MetOH. The MetOH content of juice will increase, causing a risk for consumers. Accordingly, the MetOH content in fruit and vegetable beverages should be examined to avoid a potential health hazard.

The performance of the Cu/Pt/BDD electrochemical sensor coupled with SIA in real samples was investigated by measuring the MetOH content in 10 fruit and vegetable products, and the results were compared with the reference method using gas chromatography-flame ionization detection (GC-FID). The values for the reference method were analyzed by the Food Research and Testing Laboratory, Faculty of Science, Chulalongkorn University, Thailand. The results in Table 2 indicate good agreement between the proposed method and the reference method. The statistical paired *t*-test also confirmed the absence of systematic differences between the results of these methods (*t*_observed_ = 0.09 and *t*_critical_ = 2.04 at P = 0.05). The spiked samples at 5 and 10 mM were also analyzed. Good recoveries of 97–106% were obtained. These results suggest that our proposed method can be applied to detect MetOH in fruit and vegetable beverages.

**Table 2:** Determination of methanol in fruit and vegetable beverages by the proposed method and reference method.

Sample	Added (mM)	This work		GC-FID^a^	
		Found ± SD (mM)	Recovery (%)	Found (mM)	Recovery (%)
Apple juice	0	ND	–	ND	–
	5	5.06 ± 0.08	101	4.93	99
	10	10.54 ± 0.10	105	9.71	97
Tomato juice	0	ND	–	ND	–
	5	5.10 ± 0.01	102	4.93	99
	10	10.63 ± 0.09	106	10.21	102
Orange juice	0	0.89 ± 0.02	–	0.92	–
	5	5.88 ± 0.11	100	5.93	100
	10	10.87 ± 0.03	100	11.61	106
Pineapple juice	0	1.72 ± 0.01	–	1.70	–
	5	6.79 ± 0.01	101	6.73	100
	10	11.75 ± 0.10	100	11.68	100
Green tea	0	ND	–	ND	–
	5	4.98 ± 0.01	100	5.25	105
	10	10.18 ± 0.01	102	10.33	103
Coffee	0	ND	–	ND	–
	5	5.02 ± 0.07	100	5.03	101
	10	10.17 ± 0.01	102	9.93	99
Malt beverage	0	0.89 ± 0.07	–	0.88	–
	5	5.82 ± 0.01	99	5.91	101
	10	10.89 ± 0.01	100	11.14	102
Pickled ginger	0	ND	–	ND	–
	5	4.98 ± 0.01	100	5.22	104
	10	10.18 ± 0.01	102	10.12	101
Pickled mustard	0	0.52 ± 0.00	–	0.53	–
	5	5.58 ± 0.00	101	5.65	102
	10	10.54 ± 0.07	100	10.67	101
Pickled lettuce	0	0.95 ± 0.05	–	0.92	–
	5	5.95 ± 0.01	100	6.17	104
	10	10.97 ± 0.07	100	10.86	99

^a^Food Research and Testing Laboratory, Faculty of Science, Chulalongkorn University.

## Conclusions

In summary, the Cu/Pt/BDD electrochemical sensor was successfully developed by sequential electrodeposition. The order of metal deposition and the electrodeposition technique were key factors for MetOH oxidation performance. Both the morphology and electronic coupling effect between Cu and Pt components have a significant effect on the electrochemical properties, promoting the oxidation of MetOH. While Pt showed specific electrocatalytic activity, Cu increased the number of active sites, enhanced the conductivity, and provided long-term stability. In particular, the BDD electrode acted as an efficient supporting material for the effective dispersion of metal catalysts with high robustness and electrochemical properties. Moreover, the combination of the Cu/Pt/BDD electrochemical sensor with the SIA system can be useful for MetOH detection in fruit and vegetable beverages.

## Materials and methods

### Chemicals

Copper(II) acetate (Cu(CH_3_COO)_2_) was a product of Carlo Erba reagent, France (http://www.carloerbareagents.com). Potassium tetrachloroplatinate(II) (K_2_[PtCl_4_]) and other chemical reagents were purchased from Merck, Germany (http://www.merckmillipore.com). All reagents were of analytical reagent grade and prepared using ultrapure water (resistivity ≥ 18.2 MΩ cm), obtained from a Milli-Q Ultrapure Water Purification System (Millipore, USA). BDD electrode (10,000 ppm of boron doping, 12 × 12 mm square) was obtained from NeoCoat (http://www.neocoat.ch, Switzerland) for using as the working electrode. The conductive inks from Gwent Group (http://www.gwent.org, UK) were used to construct the screen-printed electrode. Ag/AgCl ink was used as conducting pads and reference electrode, whereas carbon ink was used as the counter electrode. Polyurethane ink (Chaiyaboon, Thailand, http://www.chaiyaboon.com) was employed as the insulator.

### Apparatus and instrument

A sequential injection system (MGC Auto‐Pret MP‐014S, MGC, Japan) consisted of an 8‐port selection valve, a 5.0 mL syringe pump and a 5.0 mL holding coil was used to control and transfer chemical reagents. The electrochemical measurements were carried out in a cross-flow cell (MF-1093, Bioanalytical Systems Inc., USA) comprising of a 1.0-mm-thick silicone gasket as a spacer and a three-electrode system. All measurements were performed using a potentiostat (PGSTAT 30, Metrohm Autolab B.V., The Netherlands). The scanning electron microscope (SEM; S-3400N, Hitachi, Japan) was used to characterize the surface morphology of the working electrode surface. X-ray photoelectron spectroscopy (XPS; Ultra Axis, Bara Scientific, Thailand) was used to analyze the valence states and surface compositions.

### Electrochemical sensor fabrication

The screen-printed electrode consisted of Ag/AgCl and carbon inks to be employed as a reference electrode and a counter electrode, respectively. These electrodes were fabricated on a PVC substrate (0.15 mm thick) using an in-house screen-printing technique, and the working area was controlled by covering the surface with a polyurethane layer^38^. The center of these two electrodes was punched to form a circular hole with a diameter of 0.3 cm. Acrylic double-sided adhesive was used to attach the screen-printed electrode with a circular hole onto the BDD electrode. The obtained electrochemical sensor consisted of a Ag/AgCl reference electrode, a carbon counter electrode, and a BDD working electrode with a geometric area of 0.07 cm^2^.

### Electrode modification

To reduce the BDD background current, prior to modification, potentiostatic anodic polarization in 0.1 M H_2_SO_4_ at 2.0 V for 5 min was initially performed to eliminate the adsorbed hydrogen and the sp^2^ graphitic phase^39^. For electrode modification, K_2_[PtCl_4_] and Cu(CH_3_COO)_2_ solutions were sequentially electrodeposited on the treated BDD electrode. First, an aliquot of 1 mM K_2_[PtCl_4_] (100 µL) in 0.1 M H_2_SO_4_ was thoroughly dropped on the three-electrode surface, followed by multiple-step electrodeposition using a deposition potential of − 0.4 V and relaxation potential of 0.0 V for 600 s. The residual and undeposited particles were removed by rinsing with ultrapure water. After completing these steps, a Pt/BDD electrode was acquired. After that, 1 mM Cu(CH_3_COO)_2_ in 0.1 M CH_3_COONa (100 µL) was subsequently electrodeposited onto the cleaned and dried Pt/BDD electrode by applying a deposition potential of − 0.5 V for 900 s. The modified electrodes were then thoroughly washed with ultrapure water, and a bimetallic Cu–Pt nanocatalyst electrode (Cu/Pt/BDD) was finally achieved.

### Electrochemical measurement

The electrochemical experiment for MetOH oxidation was performed in 0.1 M NaOH as the optimized supporting electrolyte at room temperature (25 °C). Cyclic voltammetry (CV) was first performed at a scan rate of 100 mV/s to study MetOH oxidation behavior. When combined with the SIA system, 100 µL of sample was transferred to an electrochemical cell with a flow rate of 100 µL/s. Then, chronoamperometry was carried out using an applied potential of 0.5 V vs Ag/AgCl. A calibration plot was constructed to assess the detection limit and sensitivity.

## Supplementary Information

Supplementary Information.
