# Supplementary material for: Sequential electrodeposition of Cu–Pt bimetallic nanocatalysts on boron-doped diamond electrodes for the simple and rapid detection of methanol
Source: Sci Rep. 2021 Jul 13;11:14354. doi: 10.1038/s41598-021-92769-w (PMC8277777; doi:10.1038/s41598-021-92769-w)
Supplement: Supplementary file 1 — Supplementary Information. [file 41598_2021_92769_MOESM1_ESM.pdf]

Supplementary Material

Sequential electrodeposition of Cu-Pt bimetallic nanocatalysts on boron-doped diamond electrodes for the simple and rapid detection of methanol

Surinya Traipop[a], Abdulhadee Yakoh[a,b], Sakda Jampasa[a,b], Sudkate Chaiyo[a,b], Yuttanant Boonyongmaneerat[c], Joongjai Panpranot[d], Piyasan Praserthdam[d], Orawon Chailapakul[a,]*

[a] Electrochemistry and Optical Spectroscopy Center of Excellence (EOSCE), Department of Chemistry, Faculty of Science, Chulalongkorn University, Bangkok 10330, Thailand.

[b] The Institute of Biotechnology and Genetic Engineering, Chulalongkorn University, Bangkok 10330, Thailand.

[c] Metallurgy and Materials Science Research Institute, Chulalongkorn University, Bangkok 10330, Thailand.

[d] Center of Excellence on Catalysis and Catalytic Reaction Engineering, Department of Chemical Engineering, Faculty of Engineering, Chulalongkorn University, Bangkok, 10330, Thailand.

*Corresponding author: Prof. Dr. O. Chailapakul

E-mail: corawon@chula.ac.th

Tel.: +66 2 218 7615

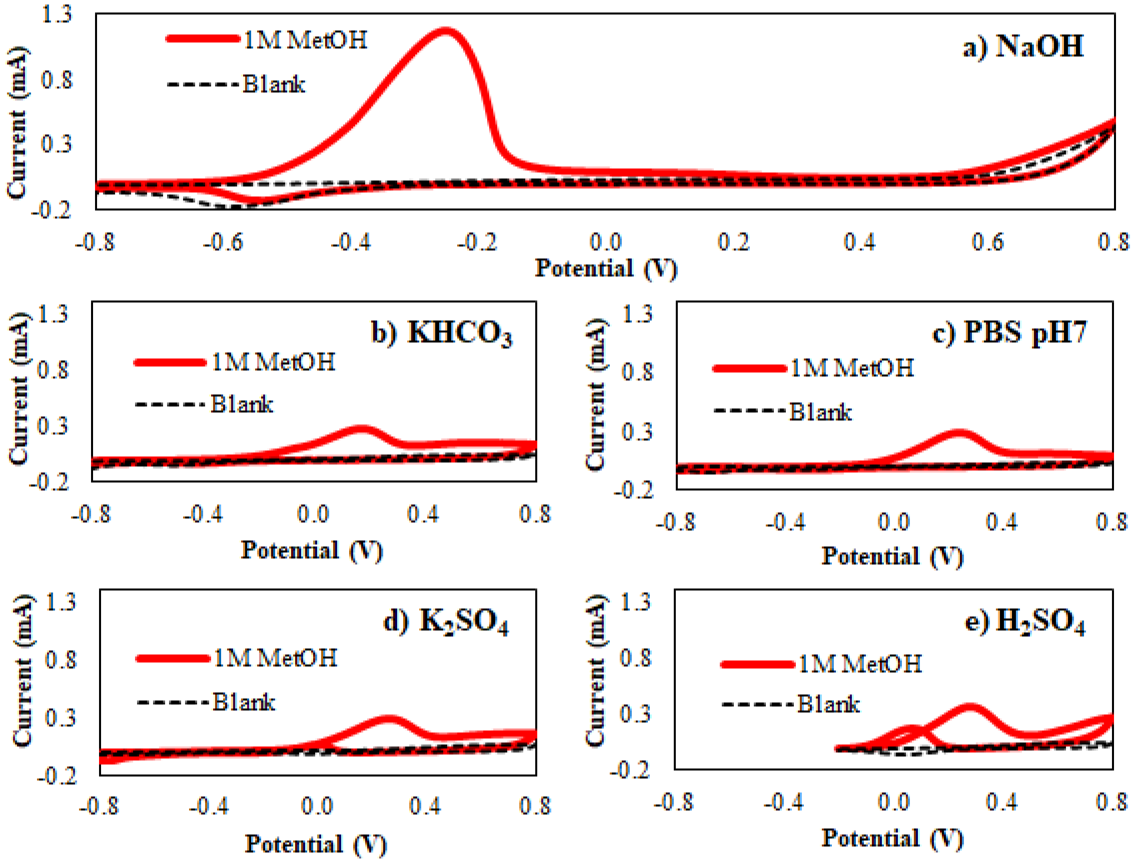

Fig.S1 Cyclic voltmmogram of 1 M methanol containing 0.1 M of a) NaOH, b) $KHCO_3$, c) PBS pH7, d) K_2SO_4, and e) H_2SO_4 on Pt/BDD electrode, Scan rate: 100 mV/s.

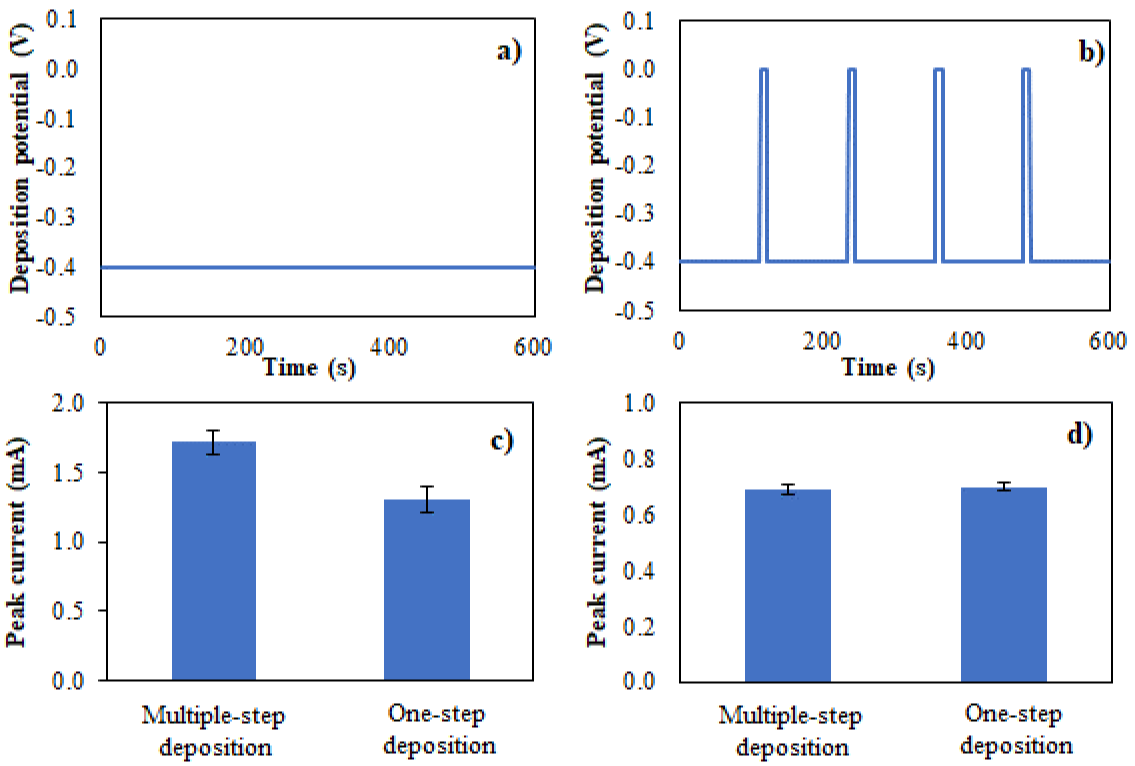

Fig.S2 Potential-time waveform of a) one-step and b) multiple-step electrodepositions; comparison of peak current obtained from methanol oxidation on a) Pt/BDD and b) Cu/BDD electrodes.

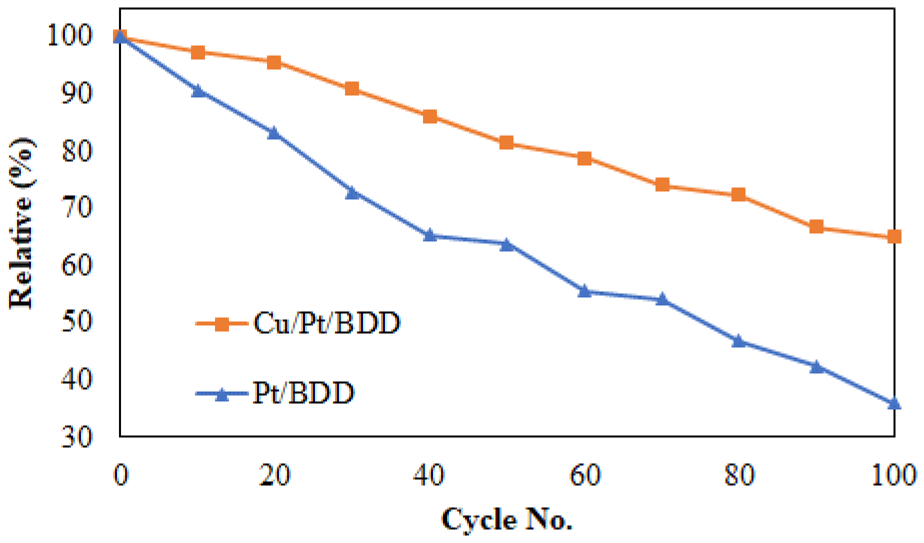

Fig.S3 The relative of MetOH oxidation on Cu/Pt/BDD electrode compared with Pt/BDD electrode.

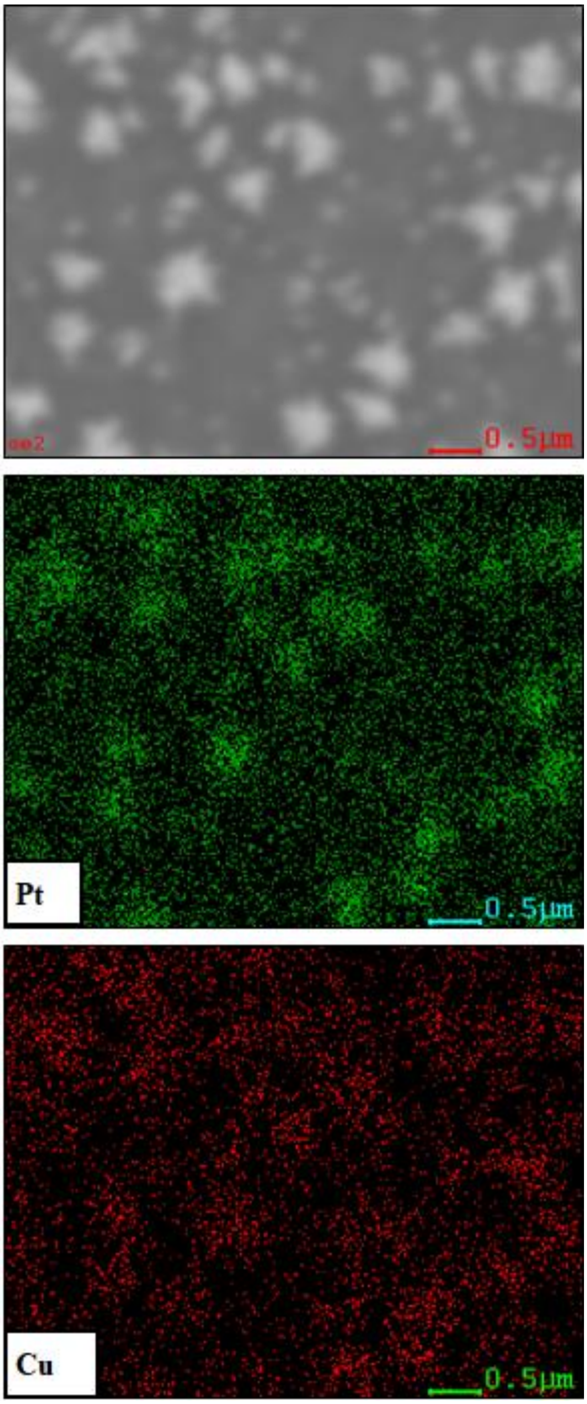

Fig.S4 SEM image and its corresponding EDS mapping of Pt and Cu on Cu/Pt/BDD electrode.

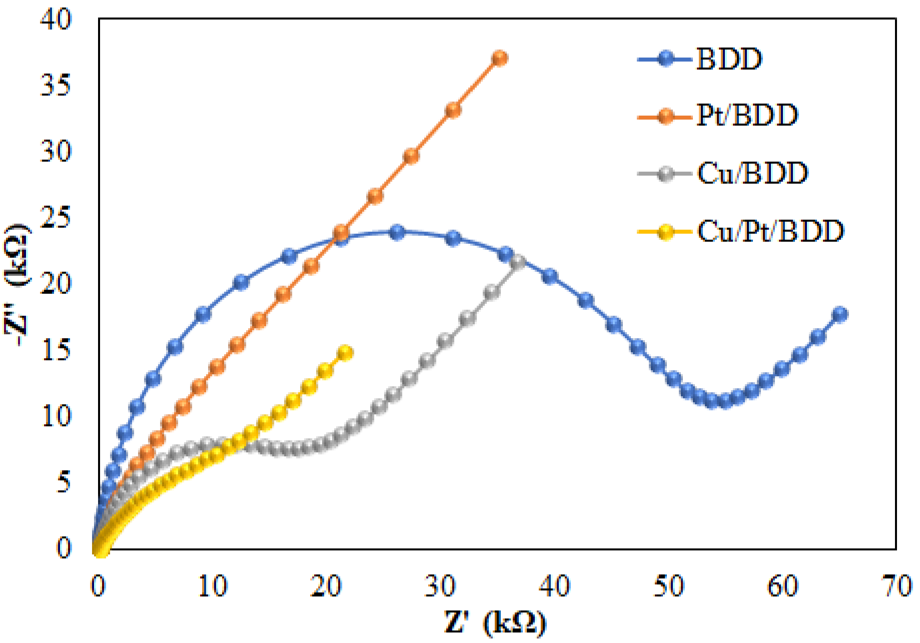

Fig.S5 Nyquist plots in the frequency range of 10-100 kHz recorded at BDD, Pt/BDD, Cu/BDD, and Cu/Pt/BDD electrodes in a solution of 0.5 M KCl containing 1 mM $[Fe(CN)_6]^{3-/4-}$.

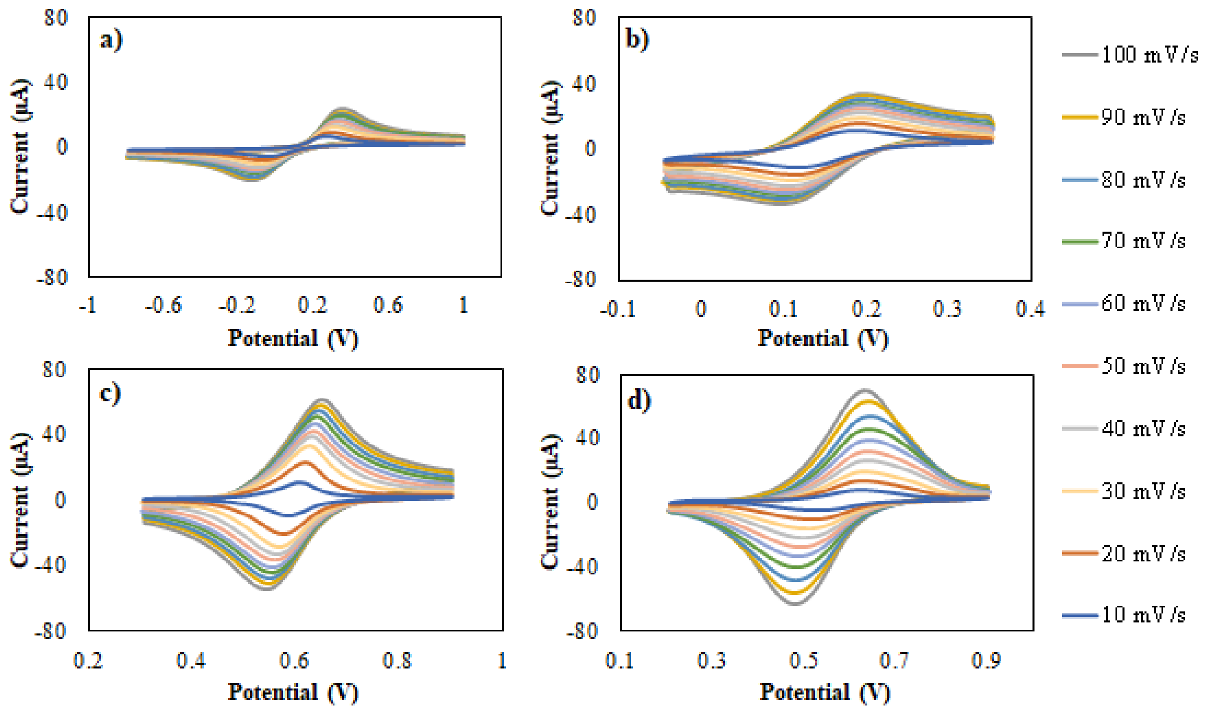

Fig.S6 cyclic voltammogram of 1 mM $[Fe(CN)_6]^{3-/4-}$ in 0.5 M KCl at various scan rates on a) BDD, b) Pt/BDD, c) Cu/BDD, and d) Cu/Pt/BDD electrodes.

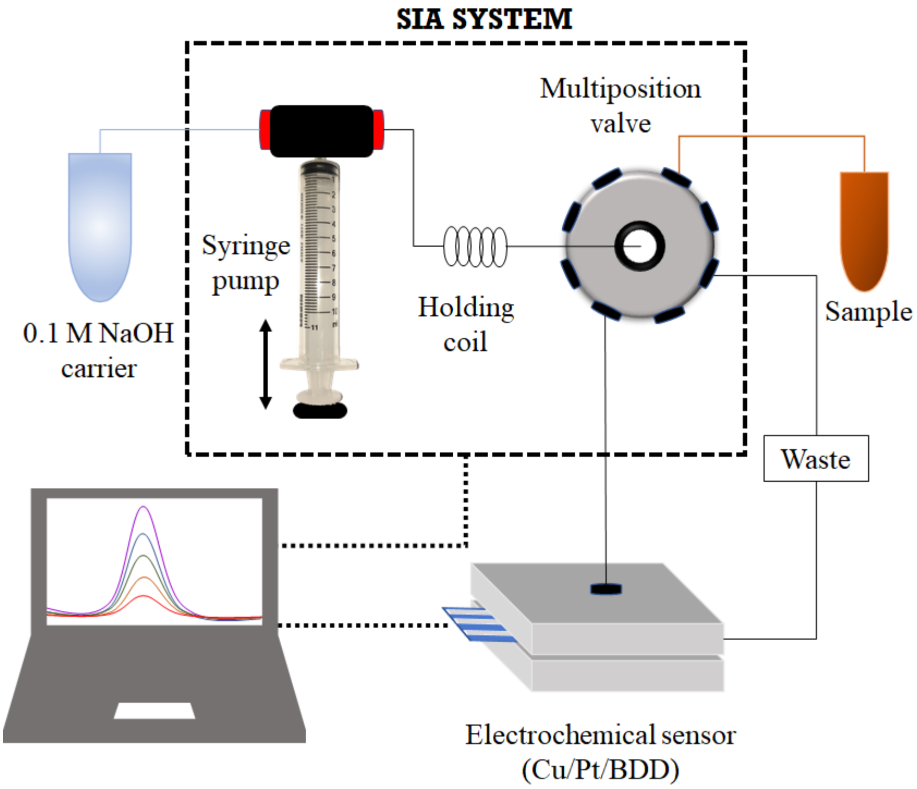

Fig.S7 Diagram of Cu/Pt/BDD electrode coupled with SIA for methanol detection.

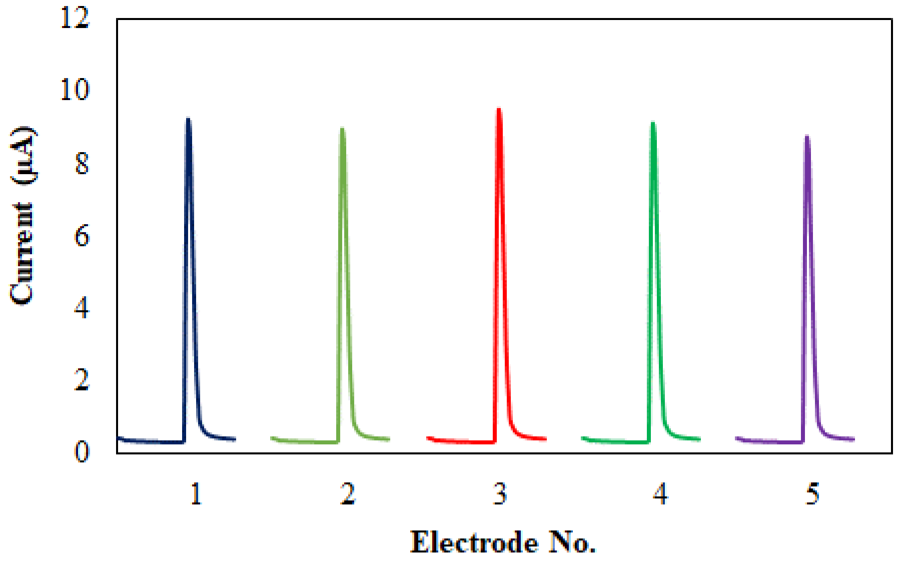

Fig.S8 Chronoamperometric responses of 10 mM MetOH on five independently fabricated Cu/Pt/BDD electrodes.

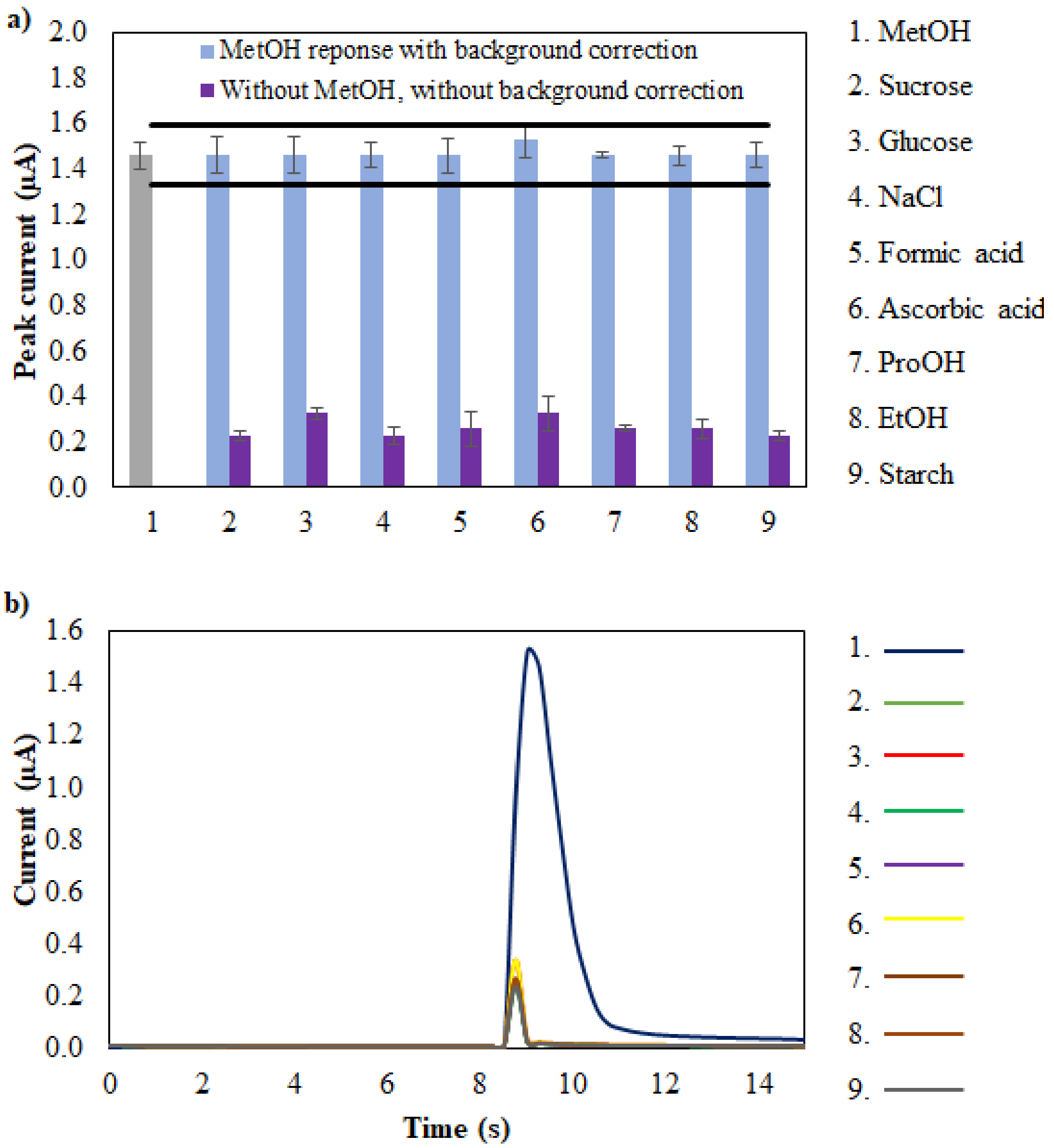

Fig.S9 a) Methanol oxidation signal derived from chronoamperogram in the presence of interferences with background correction compared with interferents signal without background correction; b) chronoamperogram of the individual studied interferents.

Table S1 The electrochemical characteristics of the modified electrodes.

**Electrode**	**A_{eff} (cm^2)**	**R_{ct} (kΩ)**	**K_{et} (cm/s)**
BDD	0.095	49.0	0.66×10^{-5}
Pt/BDD	0.097	19.2	1.69×10^{-5}
Cu/BDD	0.205	16.9	1.92×10^{-5}
Cu/Pt/BDD	0.303	14.8	2.19×10^{-5}

Table S2 The experimentally determined amount and ratio of Cu and Pt for the different modified electrodes.

**Electrodes**	**Cu amount [a]**		**Pt amount [a]**		**Mass concentration [b]**	
	**(g equivalent)**	**(%)**	**(g equivalent)**	**(%)**	**Cu (%)**	**Pt (%)**
Pt/BDD*	-	0	1.54×10^{-8}	100	0	100
Pt/BDD	-	0	2.03×10^{-8}	100	0	100
Cu/BDD	1.03×10^{-8}	100	-	0	100	0
PtCu/BDD	1.65×10^{-8}	50	1.65×10^{-8}	50	22	78
Pt/Cu/BDD	1.04×10^{-8}	29	2.54×10^{-8}	71	0	100
Cu/Pt/BDD*	3.86×10^{-8}	66	2.01×10^{-8}	34	57	43
Cu/Pt/BDD	3.76×10^{-8}	66	1.95×10^{-8}	34	58	42

[a] estimated amount from deposition profile

[b] estimated mass ratio from XPS spectrum

Table S3 The estimated amounts of deposited metals; Pt on BDD electrode and Cu on Pt/BDD electrode.

**Deposition potential (V)**	**Metal amount (g equivalent)**
Pt atoms	
-0.30	$8.16 \pm 1.56 \times 10^{-9}$
-0.35	$1.25 \pm 0.22\times 10^{-8}$
-0.38	$2.11 \pm 0.24\times 10^{-8}$
-0.40	$2.49 \pm 0.76\times 10^{-8}$
-0.45	$3.05 \pm 0.45\times 10^{-8}$ *
-0.50	$2.77 \pm 1.30\times 10^{-8}$ *
Cu atoms	
-0.30	$2.05 \pm 0.08\times 10^{-7}$
-0.40	$2.14 \pm 0.05\times 10^{-7}$
-0.50	$2.43 \pm 0.03\times 10^{-7}$
-0.60	$2.31 \pm 0.12\times 10^{-7}$
-0.70	$2.32 \pm 0.02\times 10^{-7}$

*These values were interrupted by hydrogen evolution.
